# Development and validation of the English version of the Moral Growth Mindset measure

**DOI:** 10.12688/f1000research.23160.3

**Published:** 2020-07-21

**Authors:** Hyemin Han, Kelsie J. Dawson, YeEun Rachel Choi, Youn-Jeng Choi, Andrea L. Glenn

**Affiliations:** 1 Educational Psychology Program, University of Alabama, Tuscaloosa, AL, USA; 2 Educational Research Program, University of Alabama, Tuscaloosa, AL, USA; 3 Center for the Prevention of Youth Behavior Problems, University of Alabama, Tuscaloosa, AL, USA

**Keywords:** moral growth mindset, growth mindset, reliability, validity, moral development

## Abstract

**Background**: Moral Growth Mindset (MGM) is a belief about whether one can become a morally better person through efforts. Prior research showed that MGM is positively associated with promotion of moral motivation among adolescents and young adults. We developed and tested the English version of the MGM measure in this study with data collected from college student participants.

**Methods**: In Study 1, we tested the reliability and validity of the MGM measure with two-wave data ( *N* = 212, Age mean = 24.18 years, *SD* = 7.82 years). In Study 2, we retested the construct validity of the MGM measure once again and its association with other moral and positive psychological indicators to test its convergent and discriminant validity ( *N* = 275, Age mean = 22.02 years, *SD* = 6.34 years).

**Results**: We found that the MGM measure was reliable and valid from Study 1. In Study 2, the results indicated that the MGM was well correlated with other moral and positive psychological indicators as expected.

**Conclusions**: We developed and validated the English version of the MGM measure in the present study. The results from studies 1 and 2 supported the reliability and validity of the MGM measure. Given this, we found that the English version of the MGM measure can measure one’s MGM as we intended.

## Introduction

In the present study, we aimed to create and validate the English version of the Moral Growth Mindset (MGM) measure, which was originally developed in Korean. *Growth mindset* refers to the belief that it is possible to improve one’s abilities and qualities, such as intelligence or personality ^1^. These individuals believe that this can be done through effort and learning, which helps fosters motivation. Higher motivation for those with a growth mindset is encouraged through having attitudes such as viewing hardships as a chance to work harder rather than an indication of failure, and striving for success due to genuinely wanting to learn instead of being concerned with how others view them ^2^. One study found that an intervention that taught students how to endorse a growth mindset reduced levels of aggression as well as depressive symptoms that resulted from being a victim of bullying ^3^. This study suggested that growth mindset might be beneficial for promoting a sense of resilience when faced with social challenges or other difficulties.

*MGM* refers to growth mindset in the domain of morality. This mindset is related to one’s belief that it is possible to become a morally better person and improve one’s morals through efforts. A previous study showed that MGM was positively associated with increases in voluntary service engagement among adolescents and young adults ^4^. The results suggested that among younger populations, MGM might increase participants’ prosocial behavior due to the belief that it will make them morally better. Given this, MGM would be considered as a factor that contributes to moral development. In order to adequately examine how MGM contributes to moral development, however, it is necessary to have an appropriate measure. Additionally, if moral growth mindset motivates people to learn how to become more moral, as previous research suggests, then it is important for moral educators to have a tool to assess the malleability beliefs students have related to their morals. For example, if moral educators are able to identify that some students have a fixed mindset related to their morals, then an appropriate starting point may be to provide them with evidence that it is possible to improve moral character throughout one’s life.

Although how MGM influences moral growth among people have been examined in several previous studies with a quantitative measure (e.g., 1, 5, 6), we found some points that could be improved in them, so we intended to develop and test our updated MGM measure for future studies. MGM was previously included as a three-item subscale in a general measure of growth mindset called the Theory Measures ^5, 6^. However, because it is important to include four or more items per factor to perform psychometric tests ^7^, the psychometrical qualities of the MGM subscale could not be sufficiently tested. For instance, the aforementioned previous studies examined the MGM as a subscale, so they could not sufficiently examine its internal structure and its association with diverse moral and positive psychological indicators. In a previous study ^4^, we developed and tested a Korean version of the MGM measure and evaluated the internal consistency and structure of the measure. However, the test-retest consistency and discriminant validity of the measure were not examined. Hence, in the present study, we created an English version of the MGM measure and tested its psychometric properties. In Study 1, we tested the internal and test-retest consistency and validity of the MGM measure and modified the measure to improve the model fit. In Study 2, we examined correlations between the MGM and other moral and positive psychological indicators associated with positive youth development to test the convergent and discriminant validity of the measure.

## Study 1

In Study 1, we translated the MGM measure to English and tested its reliability and validity with two-wave data. We also modified the items to improve the model fit.

### Methods

***Translation of the MGM measure to English.*** Based on the Korean version of the MGM measure ^4^ and the Implicit Theory measure ^1, 8^, we developed the English version of the MGM measure. Although the English version was created based on the Korean version, we did not do direct translation because of cultural differences in concepts and terms related to morals and characters (e.g., 9). Instead, the inventors (HH, KJD, and YJC) of the Korean MGM measure created its English version based on the structure of the Korean version and the wording in the Implicit Theory measure. In addition, the Implicit Theory measure was used due to the fact that it had six items and was based on Dweck’s original measure of growth mindset for intelligence. As a result, the tested measure included six items as well (e.g., “No matter who you are, you can significantly improve your morals and character”) and answers were anchored to a six-point Likert scale (i.e., strongly disagree (1), disagree (2), mostly disagree (3), mostly agree (4), agree (5), strongly agree (6)) (see *Extended data* for the full measure ^10^).

Although Chiu, Hong, and Dweck ^11^ originally used more nuanced keywords such as “responsible and sincere” as well as “conscientiousness, uprightness, and honesty,” we decided to use the more general terms, “morals and character.” This was due to the concern that such nuanced terms in the original measure may be associated with specific moral foundations and biased towards certain groups of people. For example, conservatives have been found to score higher on measures of conscientiousness ^12^ whereas liberals have been found to rely primarily on the value of fairness, which is closely related to honesty, when dealing with moral issues (see research on Moral Foundation Theory; e.g., 13). Thus, we used “morals and characters” in order for participants to be able to define the terms based on their own experiences and understanding. Finally, since Chiu *et al.* (1994) ^11^ used terms related to specific morals and characteristics in their original three-item subscale (e.g., “A person’s moral character,” “whether a person is responsible and sincere,” “a person’s moral traits”), we decided to use “morals and character” in order to stay consistent with the construct they were measuring. That is, rather than measuring participants’ malleability beliefs about the overarching system of values they have, we wanted to measure malleability beliefs regarding individual morals, as did the original measure. Doing so may increase the chance for interventions since if people want to become a better person (improve their morality) they may need to believe that their values (morals) can be improved.

There might be another concern regarding use of the terms, “morals and characters,” in our measure related to whether a simple belief about the possibility to improve ones’ morals (or moral values) is directly relevant to their moral growth. For instance, one previous study about the relationship between one’s endorsed personal values and behavioral outcomes reported that the correlation between values and behaviors, particularly other-reported behaviors, was moderate at the greatest ^14^. Despite of this potential issue, however, we decided to use the terms because we tried to design the measure so that participants intuitively perceive and interpret items about “morals and characters” while considering their own beliefs about moral growth. In fact, researchers in implicit theories proposed that both incremental and entity theories are intuitive and “implicit” to people ^15^, so we intended to develop our items based on this point.

Our measure is in line with the original measure, the Implicit Theory measure, consisting of six items ^1^. In fact, although all of the items were meant to measure whether or not participants endorse a growth mindset and are similar to each other, the wordings varied slightly to include core concepts of growth mindset such as being able to improve regardless of who you are (i.e., “no matter who you are”), the point in time (i.e., “always”), or the degree (i.e., “considerably”). In addition, because we were interested in whether MGM can be differentiated from growth mindset in general measured by the original growth mindset measure, we decided to use the same terms and format that were adopted in the original measure (e.g., “No matter who you are, you can change your intelligence a lot”).

***Participants.*** Study 1 was conducted during the 2018 fall semester. Participants were recruited from students enrolled in undergraduate educational psychology classes and they were provided with a course credit. Only students who were at least 18 years of age were eligible to complete the survey. The participants visited the subject pool system, checked the list of active research projects, and selected and signed up for our study. We decided to recruit at least 200 participants since *N* = 200 has been regarded as the recommended minimum sample size for confirmatory factor analysis (CFA) ^16^.

A total of 212 college students (89.15% females; Age mean = 24.18 years, *SD* = 7.82 years; 177 Caucasian, 34 African American, 1 Native American, 1 Asian, 1 Pacific Islander, 3 Latinx, 2 multi-ethnic) from the southern USA completed the English MGM measure online via Qualtrics. They were re-invited to complete the same survey again one week later ( *N* = 207 for Wave 2; 89.37% females; Age mean = 24.28 years, *SD* = 7.88 years).

***Procedures.*** Participants who voluntarily signed up for study 1 received a link to the Qualtrics survey where they completed the MGM measure, followed by a demographics survey. When the participants signed up for the study, the subject pool manager provided us with their email addresses, and we sent the participants the survey link via email. We created our Qualtrics survey in a way that only the participants who answered all survey questions were able to complete the survey and receive a credit for their class. Thus, there was no missing data in the present study.

A consent form was sent out to the students alongside the MGM measure. This form was reviewed by the Institutional Review Board at the University of Alabama (IRB approval number: 18-04-1156), along with the approved studies, and was presented at the beginning of the Qualtrics form. Only students who read the form and agreed to participate in this study were presented with the survey forms.

***Analysis.*** When examining test-retest reliability, we excluded participants who failed to complete the second survey within two weeks to control for the time gap between the two surveys, which left 168 cases for examining test-retest reliability (Mean time gap between Waves 1 and 2 = 7.78 days, *SD* = 1.66 days).

First, we examined consistency indices, i.e., Cronbach’s α and test-retest consistency. Second, we performed CFA to examine the internal structure of the measure. We used robust weighted least squares (WLSMV) because it is more suitable for testing Likert-type items in a small sample ^17^. During this process, we checked whether any item should be excluded from the measure to achieve a good model fit. If the measure was modified, we calculated all reliability and validity indices again. We used R (3.6.1) for statistical analyses. All data files and source codes are available as *Underlying data* ^10^.

### Results

First, the measure demonstrated at least acceptable reliability (> .7; see Table 1) according to both Cronbach’s alpha values and test-retest reliability. Second, we performed CFA – the original model with all six items did not show good model fit (see Table 1). Thus, we excluded items 1 and 2 while referring to Han *et al.* (2018), because in that study we showed relatively lower factor loadings in the six-item and five-item models respectively. In the supplementary table in *Extended data*, we presented factor loadings for the six-item and five-item models. In the six-item model, Item 1 showed the lowest standardized factor loading, identical to what was reported in Han *et al.* (2018) ^4^. After excluding Item 1, Item 2 showed the lowest standardized loading in the five-item model, so we removed this item accordingly.

**Table 1.** MGM measure English reliability check and confirmatory factor analysis (CFA) results.

Model	Reliability		Classical CFA						
	Cronbach α	Test-retest *r*	*χ*2	*df*	*p*	CFI	TLI	RMSEA	SRMR
**Study 1**									
6-item	.86	.76	60.08	9	.000	.84	.73	.16	.09
5-item (without item 1)	.86	.74	26.73	5	.000	.92	.83	.14	.07
4-item (without items 1 and 2)	.85	.70	1.79	2	.41	1.00	1.00	.00	.01
**Study 2**									
4-item (without items 1 and 2)	.77	-	1.60	2	.45	1.00	1.00	.00	.01

The CFA demonstrated that the four-item model was the best model given excellent model fit indices (chi-square test *p*-value > .05, RMSEA and SRMR < .05, TLI and CFI > .95; see Table 2 for the best model ^18^). In addition, as shown in Table 1, when we recalculated reliability indices, Cronbach α and test-retest r, after exclusion of the items, they all remained greater than .7.

**Table 2.** Factor loadings from CFA in both studies.

	Study 1		Study 2	
Item	Unstandardized	Standardized	Unstandardized	Standardized
No matter who you are, you can significantly improve your morals and character.	.72	.69	.77	.66
To be honest, you can’t really improve your morals and character.	-.73	-.73	-.46	-.39
You can always substantially improve your morals and character.	.75	.75	.89	.81
You can improve your basic morals and character considerably.	.86	.89	.93	.94

In addition to the low factor loadings, we also decided to remove items 1 and 2 due to the fact that they may have been too vague. For example, item 1 stated “you can’t really do much” and item 2 stated “you can’t improve very much” whereas the other items used words such as “significantly improve,” “always substantially improve,” and “improve…considerably” that conveyed more specific magnitude. Using the less extreme terms in items 1 and 2 may have put the items at risk of inconsistency ^19^ since it would be easier for participants’ opinions to shift regarding whether or not you can change “much.” In addition, as another possibility, items 1 and 2 are more likely about entity beliefs, not malleability beliefs that constitute the basis of growth mindset. These items contain some words perhaps related to entity beliefs (e.g., “certain morals and characters...,” “something about you…”), so they might not directly measure the core of the growth mindset construct and showed lower factor loadings compared to the other items.

## Study 2

In Study 2, we tested the correlation between MGM and other moral and positive psychological indicators associated with positive youth development. In addition, we performed CFA for model confirmation. We aimed at testing the validity of the measure, construct, convergent, and divergent validity.

We selected several moral and positive psychological measures to test the convergent and divergent validity of the MGM measure. We employed the Implicit Theory Measure ^1^, which measures growth mindset in general, particularly intelligence growth mindset and constitutes the basis of the MGM measure, to test convergent and discriminant validity. For the selection of moral psychological measures, we referred to recent articles about psychological constructs that significantly predict prosocial and civic behavior ^20^. They proposed moral judgment ^21, 22^, moral emotion (empathy) ^23^, and moral identity ^24^ as fundamental constructs in moral functioning. We also employed the Propensity to Morally Disengage Scale to examine whether the MGM showed negative correlation with moral disengagement ^25^ since Han *et al.* (2018) ^4^ reported that MGM promotes moral engagement. In addition to the aforementioned moral psychological measures, we used the Claremont Purpose Scale as a way to examine one’s positive development in terms of flourishing ^26^, given that purpose has been regarded as a possible moral virtue for eudemonic wellbeing ^27^.

In general, according to the previous studies that examined the relationship between growth mindset, positive psychological indicators, and antisocial tendency (e.g., 28– 30), we hypothesized that the sizes of correlation coefficients between MGM and other indicators, except the intelligence growth mindset, would be between .10 (small) and .30 (medium). We discussed further details regarding the hypothesized effect size of each measure in the following sections.

### Methods

***Participants.*** As per Study 1, participants were recruited from the educational psychology and psychology subject pools during the 2019 spring semester, with similar age and class enrollment restrictions employed in Study 1, Participants in educational psychology classes visited the subject pool system, checked the list of active research projects, and selected and signed up for our study. Participants in psychology classes who intended to sign up for our study visited the SONA system, reviewed the list of active studies, and then selected and signed up for the present study.

In total, 275 college students (81.45% females; Age mean = 22.02 years, SD = 6.34 years; 223 Caucasian, 39 African American, 2 Native American, 1 Asian, 1 Pacific Islander, 5 Latinx, 4 multi-ethnic) in the Southern United States of America were recruited. The consent procedure was identical to that in Study 1 (The University of Alabama IRB approval numbers: 18-10-1633, 18-12-1842).

***Procedures.*** When participants signed up for the present study, the procedure for educational psychology students was identical to that of study 1. In the case of psychology students, they were automatically provided with a link to a Qualtrics survey via the SONA system. Participants were presented with the MGM measure and other moral and positive psychological measures, all of which were presented in a randomized order, followed by a demographics survey. Similar to Study 1, only the participants who answered all questions were able to complete the survey and receive a credit, so there was no missing data in the present study. For sample size estimation, similar to Study 1, we followed the guidelines for CFA ^16^, so we determined that at least 200 participants were required.

***Measures.*** *MGM measure.* We used the four-item MGM measure used in Study 1.

*Implicit Theory Measure*. The Implicit Theory Measure was designed to measure one’s mindset regarding whether it is possible to change and improve one’s intelligence and abilities in general ^1^. The measure consists of six items and responses are anchored to a six-point Likert scale. The structure of this measure has been tested in previous studies (e.g., 1, 8). Given that the Implicit Theory Measure measures one’s general growth mindset, we expected that it would be positively correlated with MGM. However, because the construct measured by the Implicit Theory Measure is not domain specific, we also expected that the MGM would not completely overlap with this construct (discriminant validity). Given these, the effect size of the correlation coefficient would be medium to large ( *r* = +.3 - +.5).

*Behavioral Defining Issues Test (bDIT)*. The bDIT was developed to assess development of one’s moral judgment ^21, 22^.Choi *et al.* (2019) ^21^ tested its measurement structure and psychometrical qualities and found that it did not favor any gender and it showed acceptable reliability as well as concurrent validity with the DIT-1 measure. In general, the bDIT assesses whether one can make moral judgments based on the post-conventional schema instead of focusing on social norms or one’s personal interests. It consists of three moral dilemmas and 24 questions that ask what the most important moral philosophical criterion is when solving the moral dilemmas. We used a percentile score that quantified the likelihood of utilizing the post-conventional schema. Because the bDIT measures one’s moral judgment development, we expected that MGM would be positively associated with the bDIT score.

Unlike other self-report measures, the bDIT is a behavioral measure evaluating one’s developmental level of moral judgment with behavioral responses. Previous research has shown that participants could not increase their score even if they were asked to fake higher moral judgment with the DIT ^31^. Thus, the bDIT is less susceptible to social desirability bias and can measure one’s actual moral functioning instead of self-reported qualities. Given that this is a psychological test to assess one’s moral functioning and not a self-report measure, we expected that the bDIT score would be weakly correlated with MGM ( *r* ~ +.1).

*Interpersonal Reactivity Index (IRI)*. The IRI was used to measure empathic traits, i.e., empathic concern (EC), personal distress (PD), perspective taking (PT), and fantasy scale (FS) (Davis, 1983) with 28 items. The internal structure of the measure based on the four-factor model was validated in previous studies with factor analysis (see Chrysikou & Thompson, 2016). According to Decety and Cowell’s (2014) discussion regarding the relationship between different subcomponents in the IRI and moral functioning, we hypothesized that only EC and PT, not PD and FS, would be positively correlated with MGM ^32^. Given the IRI is a self-report measure, we expected a relatively larger (small to medium) effect size of correlation, *r* = +1 - +3, compared with the bDIT.

*Moral Identity Scale (MIS)*. The MIS measures moral identity in terms of whether moral values are regarded as central to one’s self-identity ^24^. Five items measure the moral internalization subscale and six items measure the moral symbolization subscale. Aquino and Reed (2002) ^24^ also performed CFA to validate its internal structure. Given that previous research showed that moral internalization is more fundamental in predicting one’s internal moral belief and motivation ^24^, we hypothesized that only moral internalization would be significantly associated with MGM. The hypothesized effect size of the correlation would be similar to that of the correlation between MGM, EC, and PT ( *r* = +.1 - +.3).

*Propensity to Morally Disengage Scale*. The moral disengagement scale measures one’s propensity to disengage from moral behavior within morally problematic situations ^25^. It measures moral disengagement propensities for eight mechanisms (i.e., moral justification, euphemistic labeling, advantageous comparison, displacement of responsibility, diffusion of responsibility, distortion of consequences, dehumanization, attribution of blame) with eight items (one item per mechanism). We used a composite score of the eight items. The internal structure of the scale was tested with CFA by Moore *et al.* (2012) ^25^. As Bandura (2002) proposed ^33^, moral disengagement is negatively associated with motivation for moral engagement. Thus, we expected moral disengagement would be negatively associated with MGM while the effect size of the correlation would be similar to the cases of the IRI and MIS (small to medium; *r* = -.1 - .3).

*Claremont Purpose Scale (CPS)*. This 12-item scale quantitatively measures purpose among adolescents using three subscales: meaningfulness, goal orientation, and beyond-the-self dimension ^26^. CPS scores were positively associated with various moral and positive psychological indicators (e.g., purpose in life, satisfaction with life, empathic concern, wisdom) in prior research ^26^. We used both the total CPS and subscale scores given that Bronk et al. (2018) ^26^ validated it with hierarchical CFA. Given previous studies that examine the association between morality, meaning ^34^, and purpose ^33, 35^, similar to the cases of the IRI and MIS, we hypothesized a small to medium effect size of the correlation between MGM and CPS ( *r* = +1 - +3).

***Analysis.*** First, we performed CFA with the MGM data again to test the internal structure of the MGM measure (construct validity). Second, we conducted correlation analyses to examine how MGM was associated with other moral and positive psychological indicators (convergent validity). Third, we tested whether or not the MGM measure examines a construct independent from intelligence growth mindset (discriminant validity) using the Fornell-Larcker criterion ^36^.

We also used R in Study 2. All data files and source codes are available as *Underlying data* ^10^.

### Results

The results of the reliability check showed that the MGM measure as well as all other measures possessed at least acceptable reliability (> .7; see Table 3). Moreover, CFA supported good internal structure of the MGM measure (see Table 1 and Table 2). However, it should be acknowledged that Item 4 showed a lower factor loading in Study 2 compared with Study 1, although the overall model fit indices were excellent. It would be an issue since researchers have regarded .40 as the threshold for a good factor loading ^37^. Although the issue could not be resolved completely with the current dataset, one larger dataset ( *N* = 701 as of May 2020) that is currently being collected for the next research project was analyzed as a possible way to address the issue. When we conducted preliminary CFA with the new dataset, all four factor loadings were greater than .75 while the CFA model showed good model fit, RMSEA = .08, SRMR = .01, TLI = .98, CFI = .99. Given this, the small factor loading of Item 4 reported in Study 2, -.39, could be addressed in the long term with additional data collection and analysis.

**Table 3.** Descriptive statistics, Cronbach’s α, and correlation test results.

	*M*	*SD*	1	2	3	4	5	6	7	8	9	10	11	12	13	14
1	4.77	.86	.77													
2	4.36	1.03	.37 ***	.90												
3	51.23	21.14	.11 †	.15 *	.78											
4	3.84	.68	.25 ***	.22 ***	.18 **	.78										
5	2.76	.66	-.06	.04	-.07	.02	.70									
6	3.61	.62	.26 ***	.14 *	.23 ***	.58 ***	-.03	.70								
7	3.47	.78	.16 *	.22 ***	.19 **	.33 ***	.22 ***	.18 **	.77							
8	4.44	.72	.34 ***	.25 ***	.20 **	.53 ***	-.09	.38 ***	.23 ***	.80						
9	3.31	.85	.04	.08	-.15 *	.09	.14 *	.13 *	.08	.10	.86					
10	2.40	1.09	-.24 ***	-.28 ***	-.15 *	-.34 ***	.11 †	-.22 ***	-.14 *	-.37 ***	-.07	.88				
11	3.83	.63	.16 **	.16 **	.01	.27 ***	-.16 **	.25 ***	.15 *	.24 ***	.23 ***	-.13 *	.89			
12	3.52	.94	.06	.07	-.13 *	.05	-.23 ***	.09	.05	.06	.24 ***	-.02	.82 ***	.90		
13	4.00	.70	.22 ***	.17 **	.08	.22 ***	-.10	.17 **	.16 **	.21 ***	.07	-.12 †	.79 ***	.49 ***	.86	
14	3.96	.77	.12 *	.16 **	.10	.40 ***	-.02	.34 ***	.16 **	.34 ***	.21 ***	-.19 **	.74 ***	.35 ***	.45 ***	.86

*Note*. *M*: mean. *SD*: standard deviation. *r*: Pearson correlation coefficient. Cronbach αs are also reported (on the diagonal).

† *p* < .10

* *p* < .05, ** *p* < .01, *** *p* < .001. 1: MGM, 2: intelligence growth mindset, 3: bDIT, 4: IRI EC, 5: IRI PD, 6: IRI PT, 7: IRI FS, 8: moral internalization, 9: moral symbolization, 10: moral disengagement, 11: CPS all, 12: CPS meaningfulness, 13: CPS goal orientation, 14: CPS beyond-the-self dimension.

Correlation analysis demonstrated a positive association between MGM, intelligence growth mindset, and other moral psychological indicators such as empathic concern, perspective taking, moral internalization, and purpose. Indicators relatively less relevant to morality, such as personal distress, symbolization, and meaningfulness, did not show a significant correlation (see Table 3). The effect size of the correlation coefficient between MGM and bDIT was small as predicted, but the correlation was non-significant ( *p* = .08). MGM was not significantly correlated with PD and CPS meaning. The correlation between MGM and moral disengagement was significantly negative. We found that the correlation coefficient between MGM and intelligence growth mindset ( *r* = .37) was smaller than the square root of the average variance extracted (AVE=.84), which indicates MGM showed discriminant validity from intelligence growth mindset.

## Discussion

We developed and tested the English version of the MGM measure in this study with data collected from emerging adult participants. In Study 1, we found that the four-item MGM measure possessed good consistency and internal structure. In fact, the previous studies that developed and tested measurements for diverse types of domain-specific growth mindset have shown that the measurements possessed good reliability and validity as well (e.g., 38, 39). Consistent with these previous studies, we were able to show that MGM can also be appropriately measured by a self-report measure, the English version of the MGM measure, as we intended.

In Study 2, we found that MGM was positively associated with moral and positive psychological indicators as hypothesized. Two exceptions were the significant associations between MGM and FS and the non-significant association between MGM and CPS meaning. First, FS is intended to quantify one’s tendency to expand their empathy toward imaginary beings, so the significant association with MGM indicates a tendency to broaden one’s empathy. Second, CPS meaning is about personal meaning, which does not necessarily always mean moral ^40^, so it makes sense that it would not be significantly associated with MGM. This result would suggest that the MGM measures a construct that is specifically about moral development in addition to positive youth development in general.

In the case of the bDIT, the effect size was within the hypothesized range, but the correlation was non-significant ( *p* = .08) perhaps due to the small sample size. As previously mentioned, this could also be due to the fact that the bDIT is a behavioral measure rather than a self-report measure like the MGM measure. Since the bDIT is less susceptible to social desirability bias, it may be necessary to further explore the possibility of bias in participants’ responses for the MGM measure in future studies.

In addition, moral disengagement was negatively correlated with MGM. Since moral disengagement allows people to dismiss negative feelings, they may have about behaving immorally using the eight mechanisms previously mentioned, this increases the likelihood of continuing to behave immorally. In this way, moral disengagement and MGM have somewhat reverse trajectories. As hypothesized, this suggests that MGM may promote engaging in moral behavior. In addition, since moral internalization, which has been shown to inhibit moral disengagement ^41^, was also positively correlated with MGM, it makes sense that our measure was negatively correlated with moral disengagement. If somebody has a strong sense of their morals and these values are internalized, this may help them to stay engaged with their standards and furthermore, be motivated to continue to be morally better.

Finally, we found good discriminant validity between the MGM measure and the intelligence growth mindset measure as a measure for growth mindset in general. This indicates that although the intelligence growth mindset measure and the MGM measure are measuring growth mindset related to different domains, they are measuring distinctly different constructs related to malleability beliefs (i.e., intelligence and morals, respectively). Given this, our MGM measure significantly contributes to growth mindset research by introducing a reliable and valid measure for growth mindset related to morals. 

The results from our correlation analysis are consistent with findings in previous studies that have examined the positive relationship between growth mindset and successful social adjustment and positive youth development in general ^2, 28, 42^. 

This English version of the MGM measure has the potential to significantly contribute to research in moral development and education. For instance, researchers and educators who are interested in how MGM is associated with moral development may use the MGM measure in their studies. In addition, given that we created the English version of the MGM measure, scholars who are using languages other than Korean or English will be able to translate the measure into their languages. By doing so, it would be possible to accumulate large-scale datasets for testing the measure in diverse backgrounds and contexts, and to examine the roles of MGM in moral development in the long term.

However, there are limitations in this study that warrant future studies. First, we collected data only from undergraduate students and male students were underrepresented in both studies; such issues may limit the generalizability of our findings. Second, although we used straightforward terms (e.g., morals and characters), we could not test whether the measure was actually unbiased according to one’s political orientation of endorsed moral foundations. To address this issue, measurement invariance test would be a way to examine whether the MGM measure, which allows participants to interpret “morals” and “characters” by themselves, measures the same construct across different groups who may use different underlying folk conceptions of morals and characters. Third, although participants spent about 33.98 minutes (median) to complete Study 2 we did not include any attention check items. Fourth, we did not employ Chiu et al.’s (1997) ^5^ original measure, which could be informative while conducting the convergent validity check, although our measure was based on Dweck’s (2000) ^1^ updated six-item intelligence growth mindset measure. Fifth, the items used in the MGM measure could be revised particularly when being administered among younger populations. We decided to use the current wordings to maintain consistency with the Korean version of the MGM measure and the Implicit Theory Measure, which constituted the basis of our measure. However, to make the measure more applicable to younger populations, some complex words (e.g., “substantially,” “considerably”) could be replaced with simpler words (e.g., “a lot”). Sixth, since several items in the measure might seem to be similar, the words could be revised in future studies, particularly those focusing on children or young adolescents. Although we decided to use the items overlapping with each other to make our measure consistent with previous growth mindset measures, further studies are needed to examine which form of the measure would be more appropriate. Seventh, the MGM has been tested only in the United States (mainly among Caucasians) and Korea, so it needs to be tested in more diverse countries and ethnicities to examine its cross-cultural validity.

## Data availability

Open Science Framework: Moral Growth Mindset is Associated with Change in Voluntary Service Engagement, https://doi.org/10.17605/OSF.IO/VMJUA ^10^.

### Underlying data

Folder “English version MGM” contains the underlying data and source code files that support the findings of this study:

- DISC.csv
- DISC_SONA.csv
- post.csv
- pre.csv

### Extended data

Folder “English version MGM” contains the following extended data files:

- README
- EJDP.R
- Supplementary Materials.docx- MGM measure in English and information about additional moral and positive psychological measures used in Study 2
- Supplementary Table.xlsx **-**Supplementary table reporting factor loadings from 6-item and 5-item models (contained in folder ‘English version MGM’, Supplementary table.xlsx)

Data are available under the terms of the Creative Commons Attribution 4.0 International license (CC-BY 4.0).
