# Supplementary material for: Development and validation of the English version of the Moral Growth Mindset measure
Source: F1000Res. 2020 Jul 21;9:256. Originally published 2020 Apr 14. [Version 3] doi: 10.12688/f1000research.23160.3 (PMC7309350; doi:10.12688/f1000research.23160.3)
Supplement: Peer review history

# Peer review: Reviewer response for version 3

**DOI:** 10.5256/f1000research.28163.r67622

**Published:** 2020-07-29

**Authors:** Susan Mangan

**Affiliations:** 1 Department of Psychology, Claremont Graduate University, Claremont, USA; 2 Thrive Center for Human Development, Pasadena, California, USA

This manuscript is much improved!

 Here are just a few additional points, mostly minor:

 Introduction:

- "Although how MGN influences moral growth among people HAS been examined..." (replace 'have' with "has").
- "we found some point that could be improved IN THE MEASURE..." (replace "in them" with "in the measure" as this sentence was a little bit confusing. I might suggest changing this sentence overall for clarity/brevity, something like: "Although how MGM influences moral growth has been examined in several previous studies with a quantitative measure, the current MGM measure is not psychometrically sound and a new measure is needed. Specifically, MGM was previously included..."
- I'd suggest mentioning here that the theory measures include the "implicit theory" (not just "theory measures") so that that term feels familiar when we read the methods. 

Study 1

- I can see why you might have the section "translation of the MGM measure to English" in the measure section, but I actually think this belongs in the literature review section, after the introduction. This is an important part of your argument for the items in your scale and their construction and makes the most sense before the study's description. 
- Additional concern/limitation: The last concern I have involves the negative wording of the items. I know this can't be helped at this stage, but negatively worded items can often be confusing for participants and reduce the reliability of a scale. I suggest adding this potential limitation to your discussion. See: Swain, S. D., Weathers, D., & Niedrich, R. W. (2008). Assessing three sources of misresponse to reversed Likert items. *Journal of Marketing Research*, *45*(1), 116-131 ^1^.

Thank you to the authors for giving such careful attention to our previous comments - I believe this manuscript is much stronger now! Though this study has limitations, Moral Growth Mindset is a wonderful addition to the literature and it is very exciting to see such bright scholars tackling new measures that make it possible to measure important concepts such as MGM. I'll be excited to see how the measurement items are used and developed further over time!

Is the work clearly and accurately presented and does it cite the current literature?

Partly

If applicable, is the statistical analysis and its interpretation appropriate?

Yes

Are all the source data underlying the results available to ensure full reproducibility?

Yes

Is the study design appropriate and is the work technically sound?

Yes

Are the conclusions drawn adequately supported by the results?

Partly

Are sufficient details of methods and analysis provided to allow replication by others?

Partly

Reviewer Expertise:

Positive Psychology Interventions, Emerging adulthood, Well-being, Self-determination theory (Psychological Need Satisfaction), Purpose, Gratitude, Positive Youth Development

I confirm that I have read this submission and believe that I have an appropriate level of expertise to confirm that it is of an acceptable scientific standard.

## References

[1] : Assessing Three Sources of Misresponse to Reversed Likert Items. *Journal of Marketing Research*.2008;45(1) : 10.1509/jmkr.45.1.116 116-131 10.1509/jmkr.45.1.116

# Peer review: Reviewer response for version 2

**DOI:** 10.5256/f1000research.26396.r64801

**Published:** 2020-06-30

**Authors:** Di You

**Affiliations:** 1 Psychology and Counseling Department, Alvernia University, Reading, PA, USA

The authors carefully designed two studies to test the reliability and validity of the English version of MGM, moral growth mindset. The literature review was relevant and coherent. The method section was well articulated. The results were clearly presented. The discussion was well-organized.

 One recommendation for future study: given for both studies, majorities of the participants are Caucasian. A future study may want to investigate whether the MGM is consistent across various ethnicities.

Is the work clearly and accurately presented and does it cite the current literature?

Yes

If applicable, is the statistical analysis and its interpretation appropriate?

Yes

Are all the source data underlying the results available to ensure full reproducibility?

Yes

Is the study design appropriate and is the work technically sound?

Yes

Are the conclusions drawn adequately supported by the results?

Yes

Are sufficient details of methods and analysis provided to allow replication by others?

Yes

Reviewer Expertise:

moral development; ethics

I confirm that I have read this submission and believe that I have an appropriate level of expertise to confirm that it is of an acceptable scientific standard.

# Response

**Published:** 2020-07-16

**Authors:** Hyemin Han

**Affiliations:** University of Alabama, USA

Dear Dr. You,

 Thank you very much for your positive comment and suggestion on our paper. Here is our response to your suggestion:

**Suggestion:** One recommendation for future study: given for both studies, majorities of the participants are Caucasian. A future study may want to investigate whether the MGM is consistent across various ethnicities.

**Response:** We appreciate your thought about the potential cross-cultural validity of our measure. We discussed the point in the discussion section:

*Seventh, the MGM has been tested only in the United States (mainly among Caucasians) and Korea, so it needs to be tested in more diverse countries and ethnicities to examine its cross-cultural validity.*

 We appreciate your time and consideration once again.

 Best,

 Hyemin

# Peer review: Reviewer response for version 2

**DOI:** 10.5256/f1000research.26396.r64142

**Published:** 2020-06-16

**Authors:** Kirsi Tirri, Elina Kuusisto

**Affiliations:** 1 Tampere University, Tampere, Finland; 2 Department of Education, University of Helsinki, Helsinki, Finland

The article is well written and constructed. The statistical analyses are sound and solid. Based on the results the instrument is reliable and valid.

1. However, we question what is the added value of the MGM instrument? In Dweck's (2000) previous work implicit beliefs about morality are investigated with her original instrument.
2. What is the relationship between morality and "morals and character"? What does morals mean here? What does character mean here? Why are these concepts combined? 
3. The basic rule of items is that they should measure only one issue per one item. 
4. In the limitations, author should reflect how to solve the issues mentioned above in the future studies.

Is the work clearly and accurately presented and does it cite the current literature?

Yes

If applicable, is the statistical analysis and its interpretation appropriate?

Yes

Are all the source data underlying the results available to ensure full reproducibility?

Yes

Is the study design appropriate and is the work technically sound?

Yes

Are the conclusions drawn adequately supported by the results?

Yes

Are sufficient details of methods and analysis provided to allow replication by others?

Yes

Reviewer Expertise:

school pedagogy, moral education, gifted education, talent development

We confirm that we have read this submission and believe that we have an appropriate level of expertise to confirm that it is of an acceptable scientific standard.

# Response

**Published:** 2020-07-16

**Authors:** Hyemin Han

**Affiliations:** University of Alabama, USA

Dear Drs. Kuusisto and Tirri,

 Thank you very much for your constructive comments and suggestions. They helped a lot while we were revising our manuscript. Here are our responses to your comments and suggestions:

**1. **However, we question what is the added value of the MGM instrument? In Dweck's (2000) previous work implicit beliefs about morality are investigated with her original instrument.

**Response:** We appreciate your comment about the contribution of our work. In the introduction section in the revised manuscript, we elaborated the point:

*Although how MGM influences moral growth among people have been examined in several previous studies with a quantitative measure (e.g., 1, 5, 6), we found some points that could be improved in them, so we intended to develop and test our updated MGM measure for future studies. MGM was previously included as a three-item subscale in a general measure of growth mindset called the Theory Measures 5, 6 . However, because it is important to include four or more items per factor to perform psychometric tests 7 , the psychometrical qualities of the MGM subscale could not be sufficiently tested. For instance, the aforementioned previous studies examined the MGM as a subscale, so they could not sufficiently examine its internal structure and its association with diverse moral and positive psychological indicators.*

**2. **What is the relationship between morality and "morals and character"? What does morals mean here? What does character mean here? Why are these concepts combined?

**Response:** Thanks a lot for your comment about the terms that we used. We agree with you that we used somehow general terms so they might not be ideal to examine specific concepts. However, we intended to examine one’s MGM regardless of their ideas about morality and character, we decided to use those more general terms instead of specific terms. These general terms might be more suitable to examine people’s folk and “implicit” concepts regarding their potential moral growth. In the revised manuscript, we discussed the point while responding to Reviewer 1’s related comment:

*Although Chiu, Hong, and Dweck 11 originally used more nuanced keywords such as “responsible and sincere” as well as “conscientiousness, uprightness, and honesty,” we decided to use the more general terms, “morals and character.” This was due to the concern that such nuanced terms in the original measure may be associated with specific moral foundations and biased towards certain groups of people. For example, conservatives have been found to score higher on measures of conscientiousness 12 whereas liberals have been found to rely primarily on the value of fairness, which is closely related to honesty, when dealing with moral issues (see research on Moral Foundation Theory; e.g., 13). Thus, we used “morals and characters” in order for participants to be able to define the terms based on their own experiences and understanding. Finally, since Chiu et al. (1994) 11 used terms related to specific morals and characteristics in their original three-item subscale (e.g., “A person’s moral character,” “whether a person is responsible and sincere,” “a person’s moral traits”), we decided to use “morals and character” in order to stay consistent with the construct they were measuring. That is, rather than measuring participants’ malleability beliefs about the overarching system of values they have, we wanted to measure malleability beliefs regarding individual morals, as did the original measure. Doing so may increase the chance for interventions since if people want to become a better person (improve their morality) they may need to believe that their values (morals) can be improved.*

*There might be another concern regarding use of the terms, “morals and characters,” in our measure related to whether a simple belief about the possibility to improve ones’ morals (or moral values) is directly relevant to their moral growth. For instance, one previous study about the relationship between one’s endorsed personal values and behavioral outcomes reported that the correlation between values and behaviors, particularly other-reported behaviors, was moderate at the greatest 40. Despite of this potential issue, however, we decided to use the terms because we tried to design the measure so that participants intuitively perceive and interpret items about “morals and characters” while considering their own beliefs about moral growth. In fact, researchers in implicit theories proposed that both incremental and entity theories are intuitive and “implicit” to people 41, so we intended to develop our items based on this point.*

 At the same time, as we agree your point in general, we would like to mention that we briefly discussed the point in a part of the discussion section regarding limitations:

*Second, although we used straightforward terms (e.g., morals and characters), we could not test whether the measure was actually unbiased according to one’s political orientation of endorsed moral foundations. To address this issue, measurement invariance test would be a way to examine whether the MGM measure, which allows participants to interpret “morals” and “characters” by themselves, measures the same construct across different groups who may use different underlying folk conceptions of morals and characters.*

 We plan to examine how different people differently perceive the terms and concepts in our follow-up studies.

**3.** The basic rule of items is that they should measure only one issue per one item.

 In the limitations, author should reflect how to solve the issues mentioned above in the future studies.

**Response:** We appreciate your comment regarding the items. In the limitation section, we discussed the point:

*Sixth, since several items in the measure might seem to be similar, the words could be revised in future studies, particularly those focusing on children or young adolescents. Although we decided to use the items overlapping with each other to make our measure consistent with previous growth mindset measures, further studies are needed to examine which form of the measure would be more appropriate.*

 Again, we sincerely appreciate your time and consideration.

 Best,

 Hyemin

# Peer review: Reviewer response for version 2

**DOI:** 10.5256/f1000research.26396.r63220

**Published:** 2020-05-11

**Authors:** Michael T. Warren

**Affiliations:** 1 Human Early Learning Partnership, University of British Columbia, British Columbia, Canada; 2 Psychology Department, Western Washington University, Bellingham, WA, USA

Dear Dr. Han,

 Thank you for the opportunity to review the revision of your Moral Growth Mindset article. I appreciate your thorough attention to detail, and particularly the improvements that were made in fully reporting the CFA results and providing rationales for the selection of constructs used to test convergent and discriminant validity in Study 2. Accordingly, I am approving the article, as only minor changes are further warranted. Thank you for your valuable contribution to moral science.

 I do remain skeptical about the word choice "improve your morals" found in the scale's items. My concern is that the phrase introduces unnecessary ambiguity. Are participants meant to interpret it as "improve yourself on specific moral traits" (based on your response I believe this is the intention), or are they meant to interpret it as "improve your moral values." To me, the former is directly relevant to becoming a better person, whereas the latter has very little to do with it-- we know, for example, that moral values and behaviour often show only small-to-moderate associations (e.g., Bardi & Schwartz, 2003 ^1^). Becoming a better person entails not only holding moral values but embodying the cognitive, affective, motivational, and behavioral  expressions of one's moral values. That said, it's entirely possible that I'm overthinking this and that typical survey respondents will intuitively grasp the meaning you intend. I'll leave it to you to decide whether this issue warrants further discussion.

 Beyond that, I would ask you to consider the following additional minor suggestions:

- Consider providing the MGM scale anchors either in the "Translation of the MGM measure to English" section (p. 3) or in a note beneath Table 2. I think requiring people to find the scale anchors in the "Supplementary Materials.docx" file may prove inconvenient; some future researchers may take the items from Table 2 and make up their own anchors.
- In discussing Item 4's smaller factor loading in Study 2, I don't think it's fair to say it had a " slightly lower factor loading in Study 2 compared with Study 1." A difference of -.73 vs. -.39 in standardized loadings is quite substantial. Additionally, I wonder if readers will know which item is #4, since you're referring to the second item in Table 2. This issue also pertains to other items mentioned by number throughout the document.
- Question: Is the Implicit Theory Measure in fact domain-general (as stated on p. 5) or does it measure intelligence growth mindset?
- I'm not sure what the following sentence means in the context of the model fit paragraph on p. 5: "As shown in Table 1, when we recalculated indices after exclusion of the items, they all remained greater than .7." I assume you're referring to alpha, but the context suggests you're saying the model fit indices were greater than .7, which is confusing. (It would also be confusing to say each item had an alpha of .7, since alpha pertains to the collection of items.) 

Best wishes,

 Michael T. Warren

Is the work clearly and accurately presented and does it cite the current literature?

Yes

If applicable, is the statistical analysis and its interpretation appropriate?

Partly

Are all the source data underlying the results available to ensure full reproducibility?

Yes

Is the study design appropriate and is the work technically sound?

Partly

Are the conclusions drawn adequately supported by the results?

Yes

Are sufficient details of methods and analysis provided to allow replication by others?

Yes

Reviewer Expertise:

Developmental Psychology, Moral Development, Adolescent Development, Mindfulness

I confirm that I have read this submission and believe that I have an appropriate level of expertise to confirm that it is of an acceptable scientific standard.

## References

[1] : Values and behavior: strength and structure of relations. *Pers Soc Psychol Bull*.2003;29(10) : 10.1177/0146167203254602 1207-20 10.1177/0146167203254602 15189583

# Response

**Published:** 2020-07-16

**Authors:** Hyemin Han

**Affiliations:** University of Alabama, USA

Dear Dr. Warren,

 We sincerely appreciate your invaluable comments once again. We were able to improve the quality of our manuscript thanks to your constructive comments and suggestions. Here are our responses to your new comments:

**1. **I do remain skeptical about the word choice "improve your morals" found in the scale's items. My concern is that the phrase introduces unnecessary ambiguity. Are participants meant to interpret it as "improve yourself on specific moral traits" (based on your response I believe this is the intention), or are they meant to interpret it as "improve your moral values." To me, the former is directly relevant to becoming a better person, whereas the latter has very little to do with it-- we know, for example, that moral values and behaviour often show only small-to-moderate associations (e.g., Bardi & Schwartz, 20031). Becoming a better person entails not only holding moral values but embodying the cognitive, affective, motivational, and behavioral  expressions of one's moral values. That said, it's entirely possible that I'm overthinking this and that typical survey respondents will intuitively grasp the meaning you intend. I'll leave it to you to decide whether this issue warrants further discussion. 

**Response: **Thank you very much for your concern regarding the use of the terms once again. We agree with you that the issue that you mentioned would be problematic in the long term, so we hope the issue is examined in our follow-up studies focusing on how different people differently perceive the concepts implied in the terms. In the revised manuscript, we added the point that you mentioned with additional citations:

*There might be another concern regarding use of the terms, “morals and characters,” in our measure related to whether a simple belief about the possibility to improve ones’ morals (or moral values) is directly relevant to their moral growth. For instance, one previous study about the relationship between one’s endorsed personal values and behavioral outcomes reported that the correlation between values and behaviors, particularly other-reported behaviors, was moderate at the greatest 40. Despite of this potential issue, however, we decided to use the terms because we tried to design the measure so that participants intuitively perceive and interpret items about “morals and characters” while considering their own beliefs about moral growth. In fact, researchers in implicit theories proposed that both incremental and entity theories are intuitive and “implicit” to people 41, so we intended to develop our items based on this point.*

**2. **Beyond that, I would ask you to consider the following additional minor suggestions:Consider providing the MGM scale anchors either in the "Translation of the MGM measure to English" section (p. 3) or in a note beneath Table 2. I think requiring people to find the scale anchors in the "Supplementary Materials.docx" file may prove inconvenient; some future researchers may take the items from Table 2 and make up their own anchors.

**Response: **We appreciate your suggestion. In the revised manuscript, we presented the Likert scale that we used in the measure:

*As a result, the tested measure included six items as well (e.g., “No matter who you are, you can significantly improve your morals and character”) and answers were anchored to a six-point Likert scale (i.e., strongly disagree (1), disagree (2), mostly disagree (3), mostly agree (4), agree (5), strongly agree (6)) (see Extended data for the full measure 10 ).*

**3. **In discussing Item 4’s smaller factor loading in Study 2, I don’t think it’s fair to say it had a “slightly lower factor loading in Study 2 compared with Study 1.” A difference of -.73 vs. -.39 in standardized loadings is quite substantial. Additionally, I wonder if readers will know which item is #4, since you’re referring to the second item in Table 2. This issue also pertains to other items mentioned by number throughout the document. 

**Response: **Thank you very much for your comment on the factor loading issue. In the revised manuscript, we added the point that you mentioned:

*It would be an issue since researchers have regarded .40 as the threshold for a good factor loading 42.*

 In addition, although it is somehow preliminary at this point, we did a quick analysis of the data collected from a part of our follow-up studies ( *N* = 701). The quick analysis showed that all factor loadings exceeded .75, so the aforementioned issue might be able to be addressed with further analysis with a larger dataset. Related to the policy of *F1000Research*, one good point to publish a paper in this platform is that it allows authors to post updated versions even after their paper has been approved by reviewers. So, to get the benefit from the platform policy that allows post-publication updates, we decided to briefly report the preliminary result in the revised manuscript:

*Although the issue could not be resolved completely with the current dataset, one larger dataset (N = 701 as of May 2020) that is currently being collected for the next research project was analyzed as a possible way to address the issue. When we conducted preliminary CFA with the new dataset, all four factor loadings were greater than .75 while the CFA model showed good model fit, RMSEA = .08, SRMR = .01, TLI = .98, CFI = .99. Given this, the small factor loading of Item 4 reported in Study 2, -.39, could be addressed in the long term with additional data collection and analysis.*

**4.** Question: Is the Implicit Theory Measure in fact domain-general (as stated on p. 5) or does it measure intelligence growth mindset? 

**Response: **We appreciate your comment that requests the clarification of the term. In the revised manuscript, we addressed the point:

*We employed the Implicit Theory Measure 1 , which measures growth mindset in general, particularly intelligence growth mindset, and constitutes the basis of the MGM measure, to test convergent and discriminant validity.*

**5. **I'm not sure what the following sentence means in the context of the model fit paragraph on p. 5: "As shown in Table 1, when we recalculated indices after exclusion of the items, they all remained greater than .7." I assume you're referring to alpha, but the context suggests you're saying the model fit indices were greater than .7, which is confusing. (It would also be confusing to say each item had an alpha of .7, since alpha pertains to the collection of items.) 

**Response: **Thanks a lot for your request for the clarification of the concept. In the revised manuscript, we addressed the concern:

*In addition, as shown in Table 1, when we recalculated reliability indices, Cronbach α and test-retest r, after exclusion of the items, they all remained greater than .7.*

 Again, we sincerely appreciate your comments and suggestions. They have significantly contributed to the improvement of the paper that we have been working on.

 Best,

 Hyemin

# Peer review: Reviewer response for version 1

**DOI:** 10.5256/f1000research.25564.r62237

**Published:** 2020-04-29

**Authors:** Susan Mangan

**Affiliations:** 1 Department of Psychology, Claremont Graduate University, Claremont, USA; 2 Thrive Center for Human Development, Pasadena, California, USA

Han *et al.* suggest the creation of a moral growth mindset scale, which could make a significant contribution to the growing literature on growth mindset. The initial validity and reliability of this scale appears sound, and the inclusion of moral growth mindset (MGM) to the literature base seems evident. Despite the promise of this scale and the concept it captures, the article, as written, has some room for improvement. Most notably, the items themselves may benefit from further revision, more information is needed, especially on the various types of validity discussed in the article (e.g. convergent, divergent), and the writing could be clearer and more concise throughout.

 Detailed responses:

- Lit review
  - One piece missing here is an argument for the significance of this scale. Why is moral growth mindset an important concept to measure? How does this measure add to existing literature (and existing measures on growth mindset + morality?) Were items for this scale taken from related scales (growth mindset?)
- Study 1
  - Elaborate on the “Implicit theory measure.” Be sure to say 1-2 sentences about this to help us understand its significance to the current study.
  - *Participants*
    - How were participants compensated? Class credit? Gift card? No compensation?
    - (Ok, later in the next paragraph you mention class credit – I would mention in the first paragraph that participants were offered class credit to participate).
  - To help clarify each portion of the study and make it easy for readers to find the information that wish, I would add a heading of “Procedure” that begins with the “Participants received a link to the qulatircs survey…”
  - *Analysis*
    - What is “underlying data” and where is it located? From the final sentence of your analysis section.
- Study 2
  - “In study 2, we tested THE correlation between…” (Add “the” to sentence).
  - You mention the SONA system and how participants selected studies here – make sure to also include this recruitment information in study 1. Additionally, here you include information about the order of survey scales and demographics, which is also not in study 1. In general, there is different information here than in study 1 – I would align these participant sections to include the same relevant information. I would also, again, separate this into a clear “participants” section and a clear “procedure” section.
    - Additionally: How long did it take participants to complete surveys? Were any attention check items included?
  - *Measures*
    - While these measures look great – we need to understand their inclusion. In the literature review portion of this article there should be discussion of each construct and why/how you expect it to relate to moral growth mindset. Why are these good choices for convergent validity?
    - You mention that further details are available in “extended data” but I don’t see a way to access this. Is all the relevant information explaining the connection of these scales to moral growth mindset provided there?
    - Additionally, did you test for divergent validity? Content validity? Construct validity? If not, why?
    - Ok, some indication of a test for discriminant validity is presented in analysis, but this should be listed with the other measures above. Similarly, more information is needed here about why you expect this to be divergent from moral growth mindset.
- Discussion
  - 1 ^st^ sentence: “We developed and tested….from youth participants.” These participants were emerging adults, correct? Not youth?
  - I would elaborate heavily on this first paragraph. What does it mean that moral disengagement was negatively correlated with MGM? What does it indicate if MGM and growth mindset were discriminant? As this is a measure development paper, I would include a richer discussion of what you found and what it indicates for each type of validity.
  - I’m not sure what you mean here in sentence two “In fact, the previous studies that developed and tested measurements for the mindset with diverse domains…” What is “the mindset”? Do you mean, that tested other types of domain-specific growth mindset?
  - There is a lot of information about previous studies coming up here that should also be in the introduction/literature review section. This would be great to include so we know what you are expecting before the study is run. Then, here in the discussion, you can tell us if your predictions came out as expected or not.
  - Paragraph 1 and 2 here are a little confusing. Be sure to start each paragraph with a general sentence that indicates what you found. Then, discuss each of your results that provides evidence for your statement, and conclude with a sentence that indicates to us the importance of these results. It feels like there are too many concepts included in each paragraph, and the writing is a bit confusing throughout.
  - Last paragraph “However, there are limitationS” (missing s).
- Items
  - These items all feel a bit too similar – Each asks if morality can or cannot be “improved.” Did you consider other items with different word choices? For example, even “You can always become a more moral person with better character.” Or “It is possible to grow in your character and morality.” These items are so similar that it feels hard to argue any differences between them.
  - Additionally, if possible it’s always best to include simpler words over more complicated ones. For instance “substantially” could easily be “a lot” and “considerably” could also be “a lot.” More complex words tax the participants a bit more, and may make the sentences more difficult to digest, especially for those with lower readings levels or from less educated backgrounds.
- General notes
  - There are a few places where the writing could be condensed or the use of alternate terms would improve ease of reading. For instance, an example of condensing would be: in participants, “Participants were recruited from an undergraduate subject pool. The pool consisted of students who were enrolled in educational psychology classes” could be condensed to “Participants were recruited from students enrolled in educational psychology classes.” A for instance of somewhere where alternate terms would be benefitial would be in: Results, “First, all consistency indicators indicated…” Instead of using “indicator/indicated” here I would recommend “First, all consistency indicators revealed…” Or, in the spirit of condensing/being more specific, I might suggest: First, the measure demonstrated at least acceptable reliability according to both cronbach’s alpha values and test-retest reliability.”
    - Look for these types of instances throughout the paper with an eye towards condensing repetitive language and becoming more specific.

Is the work clearly and accurately presented and does it cite the current literature?

Partly

If applicable, is the statistical analysis and its interpretation appropriate?

Yes

Are all the source data underlying the results available to ensure full reproducibility?

Yes

Is the study design appropriate and is the work technically sound?

Yes

Are the conclusions drawn adequately supported by the results?

Partly

Are sufficient details of methods and analysis provided to allow replication by others?

Partly

Reviewer Expertise:

Developmental psychology, positive psychology, emerging adulthood, positive pyschology interventions

I confirm that I have read this submission and believe that I have an appropriate level of expertise to confirm that it is of an acceptable scientific standard, however I have significant reservations, as outlined above.

# Response

**Published:** 2020-05-06

**Authors:** Hyemin Han

**Affiliations:** University of Alabama, USA

Dear Dr. Mangan,

 We sincerely appreciate your comments and suggestions on our manuscript. We found that they are very constructive and information. While revising our manuscript, we have done our best to address the concerns that you mentioned in your review report. Please find our responses to your comments below. Thank you very much for your time and consideration.

 Best,

 Hyemin Han (Corresponding author)

**Responses**

**1. **One piece missing here is an argument for the significance of this scale. Why is moral growth mindset an important concept to measure? How does this measure add to existing literature (and existing measures on growth mindset + morality?) Were items for this scale taken from related scales (growth mindset?) 

**Response**: We appreciate your comment regarding the explanation of why the measure is important. Also, we described how the items were developed. In the revised manuscript, we elaborated further details.

*“The results suggested that among younger populations, MGM might increase participants’ prosocial behavior due  to the belief that it will make them morally better. Given this, MGM would be considered as a factor that contributes to moral development. In order to adequately examine how MGM contributes to moral development, however, it is necessary to have an appropriate measure. Additionally, if moral growth mindset motivates people to learn how to become more moral, as previous research suggests, then it is important for moral educators to have a tool to assess the malleability beliefs students have related to their morals. For example, if moral educators are able to identify that some students have a fixed mindset related to their morals, then an appropriate starting point may be to provide them with evidence that it is possible to improve moral character throughout one’s life.”*

*“Instead, the inventors (HH, KJD, and YJC) of the Korean MGM measure created its English version based on the structure of the Korean version and the wording in the Implicit Theory measure. In addition, the Implicit Theory measure was used due to the fact that it had six items and was based on Dweck’s original measure of growth mindset for intelligence. As a result, the tested measure included six items as well (e.g., “No matter who you are, you can significantly improve your morals and character”) and answers were anchored to a six-point Likert scale (see Extended data for the full measure 10 ).”*

**2. **Elaborate on the “Implicit theory measure.” Be sure to say 1-2 sentences about this to help us understand its significance to the current study. 

**Response**: Thank you very much for your suggestion regarding the elaboration of the construct. We elaborated such a point in the revised manuscript:

*“Growth mindset refers to the belief that it is possible to improve one’s abilities and qualities, such as intelligence or personality 1 . These individuals believe that this can be done through effort and learning, which helps fosters motivation. Higher motivation for those with a growth mindset is encouraged through having attitudes such as viewing hardships as a chance to work harder  rather than an indication of  failure, and striving for success due to genuinely wanting to learn instead of being concerned with how others view them 2”*

**3. **How were participants compensated? Class credit? Gift card? No compensation?(Ok, later in the next paragraph you mention class credit – I would mention in the first paragraph that participants were offered class credit to participate).

**Response**: Thanks a lot for your request for the clarification of the compensation. In the revised manuscript, such a point is more clearly stated:

*“Participants were recruited from students enrolled in undergraduate educational psychology classes and they were provided with a course credit.”*

**4. **To help clarify each portion of the study and make it easy for readers to find the information that wish, I would add a heading of “Procedure” that begins with the “Participants received a link to the qulatircs survey…” 

**Response**: We sincerely appreciate your suggestion regarding the use of the “procedure” subsection. In the revised manuscript, we created the new subsection for a better structure.

**5. **What is “underlying data” and where is it located? From the final sentence of your analysis section. 

**Response**: Thank you for your comment regarding “underlying data.” “Underlying data” is a way to include supplementary materials in F1000Research. Readers can download the supplementary materials with the URL provided at the end of the main text. More specifically, a link to an open science repository is provided in the “data statement” section as per the journal guidelines.

**6. **“In study 2, we tested THE correlation between…” (Add “the” to sentence).You mention the SONA system and how participants selected studies here – make sure to also include this recruitment information in study 1. Additionally, here you include information about the order of survey scales and demographics, which is also not in study 1. In general, there is different information here than in study 1 – I would align these participant sections to include the same relevant information. I would also, again, separate this into a clear “participants” section and a clear “procedure” section.

**Response**: We appreciate your comments regarding the typo and the use of the independent subsection, “procedure.” We addressed these issues in the revised manuscript.

**7. **Additionally: How long did it take participants to complete surveys? Were any attention check items included? 

**Response**: Thanks a lot for your kind comment regarding the survey duration. Unfortunately, we could not include any attention check items in the survey form. We explained further details in the limitation section:

*“Third, although participants spent about 33.98 minutes (median) to complete Study 2 we did not include any attention check items.”*

**8. **While these measures look great – we need to understand their inclusion. In the literature review portion of this article there should be discussion of each construct and why/how you expect it to relate to moral growth mindset. Why are these good choices for convergent validity? 

**Response**: We sincerely appreciate your comment regarding the rationale for the inclusion of the additional measures. In the introduction section in Study 2, we added explanations regarding the point. In addition, while describing each additional measure, we explained the rational for the inclusion as well as the hypothesized correlation with MGM.

*“We selected several moral and positive psychological measures to test the convergent and divergent validity of the MGM measure. We employed the Implicit Theory Measure 1, which measures domain-general growth mindset and constitutes the basis of the MGM measure, to test convergent and discriminant validity. For the selection of moral psychological measures, we referred to recent articles about psychological constructs that significantly predict prosocial and civic behavior 31. They proposed moral judgment 18, 19, moral emotion (empathy) 20, and moral identity 21 as fundamental constructs in moral functioning. We also employed the Propensity to Morally Disengage Scale to examine whether the MGM showed negative correlation with moral disengagement 22 since Han et al. (2018) 4 reported that MGM promotes moral engagement. In addition to the aforementioned moral psychological measures, we used the Claremont Purpose Scale as a way to examine one’s positive development in terms of flourishing 23, given that purpose has been regarded as a possible moral virtue for eudemonic wellbeing 32.*

*In general, according to the previous studies that examined the relationship between growth mindset, positive psychological indicators, and antisocial tendency (e.g., 24– 26), we hypothesized that the sizes of correlation coefficients between MGM and other indicators, except the general growth mindset, would be between .10 (small) and .30 (medium). We discussed further details regarding the hypothesized effect size of each measure in the following sections.”*

**9. **You mention that further details are available in “extended data” but I don’t see a way to access this. Is all the relevant information explaining the connection of these scales to moral growth mindset provided there?

**Response**: Thank you for your comment regarding the “extended data.” Same to the “underlying data,” “extended data” can also be downloaded with the link provided at the end of the main text. In the revised manuscript, as we mentioned in our response to your comment above, we explained further details regarding each additional measure in the methods section in Study 2.

**10. **Additionally, did you test for divergent validity? Content validity? Construct validity? If not, why?

 Ok, some indication of a test for discriminant validity is presented in analysis, but this should be listed with the other measures above. Similarly, more information is needed here about why you expect this to be divergent from moral growth mindset.

**Response**: We appreciate your comment regarding the clarification of the tests that we conducted. In the revised manuscript, we explained further details.

*“We aimed at testing the validity of the measure, construct, convergent, and divergent validity.*

*We selected several moral and positive psychological measures to test the convergent and divergent validity of the MGM measure. We employed the Implicit Theory Measure 1, which measures domain-general growth mindset and constitutes the basis of the MGM measure, to test convergent and discriminant validity. For the selection of moral psychological measures, we referred to recent articles about psychological constructs that significantly predict prosocial and civic behavior 31. They proposed moral judgment 18, 19, moral emotion (empathy) 20, and moral identity 21 as fundamental constructs in moral functioning. We also employed the Propensity to Morally Disengage Scale to examine whether the MGM showed negative correlation with moral disengagement 22 since Han et al. (2018) 4 reported that MGM promotes moral engagement. In addition to the aforementioned moral psychological measures, we used the Claremont Purpose Scale as a way to examine one’s positive development in terms of flourishing 23, given that purpose has been regarded as a possible moral virtue for eudemonic wellbeing 32.”*

*“Given that the Implicit Theory Measure measures one’s general growth mindset, we expected that it would be positively correlated with MGM. However, because the construct measured by the Implicit Theory Measure is not domain specific, we also expected that the MGM would not completely overlap with this construct (discriminant validity). Given these, the effect size of the correlation coefficient would be medium to large (r = +.3 - +.5).”*

**11.** 1st sentence: “We developed and tested….from youth participants.” These participants were emerging adults, correct? Not youth?

**Response**: Thanks for your point regarding the correct use of the term. Yes, that is correct. So, we used “emerging adults” instead of “youth…” in the revised manuscript:

*“We developed and tested the English version of the MGM measure in this study with data collected from emerging adult participants.”*

**12.** I would elaborate heavily on this first paragraph. What does it mean that moral disengagement was negatively correlated with MGM? What does it indicate if MGM and growth mindset were discriminant? As this is a measure development paper, I would include a richer discussion of what you found and what it indicates for each type of validity.

**Response**: We appreciate your comment regarding moral disengagement. In the section that describes moral disengagement and its measure, we address the points that you mentioned:

*“Propensity to Morally Disengage Scale. The moral disengagement scale measures one’s propensity to disengage from moral behavior within morally problematic situations 22. It measures moral disengagement propensities for eight mechanisms (i.e., moral justification, euphemistic labeling, advantageous comparison, displacement of responsibility, diffusion of responsibility, distortion of consequences, dehumanization, attribution of blame) with eight items (one item per mechanism). We used a composite score of the eight items. The internal structure of the scale was tested with CFA by Moore et al. (2012) 22. As Bandura (2002)  proposed 35, moral disengagement is negatively associated with motivation for moral engagement. Thus, we expected moral disengagement would be negatively associated with MGM while the effect size of the correlation would be similar to the cases of the IRI and MIS (small to medium; r = -.1 - .3).”*

 In addition, in the discussion section, we elaborated the meaning of the negative correlation found from our analysis:

*“In addition, moral disengagement was negatively correlated with MGM. Since moral disengagement allows people to dismiss negative feelings, they may have about behaving immorally using the eight mechanisms previously mentioned, this increases the likelihood of continuing to behave immorally. In this way, moral disengagement and MGM have somewhat reverse trajectories. As hypothesized, this suggests that MGM may promote engaging in moral behavior. In addition, since moral internalization, which has been shown to inhibit moral disengagement 39, was also positively correlated with MGM, it makes sense that our measure was negatively correlated with moral disengagement. If somebody has a strong sense of their morals and these values are internalized, this may help them to stay engaged with their standards and furthermore, be motivated to continue to be morally better.”*

**13. **I’m not sure what you mean here in sentence two “In fact, the previous studies that developed and tested measurements for the mindset with diverse domains…” What is “the mindset”? Do you mean, that tested other types of domain-specific growth mindset?

**Response**: Thank you for your request for the clarification. Yes, that is correct. In the revised manuscript, we specified the nature of the mindset:

*“In fact, the previous studies that developed and tested measurements for diverse types of domain-specific growth mindset have shown that the measurements possessed good reliability and validity as well (e.g., 29, 30).”*

**14.** There is a lot of information about previous studies coming up here that should also be in the introduction/literature review section. This would be great to include so we know what you are expecting before the study is run. Then, here in the discussion, you can tell us if your predictions came out as expected or not.

**Response**: We sincerely appreciate your suggestion regarding rewriting the introduction. We agree with you that some theoretical contents that were presented in the discussion section in the original manuscript could be moved on to the introduction section for a better structure. Following your suggestion, in the revised manuscript, we presented such contents in the general introduction or introduction of each study.

**15. **Paragraph 1 and 2 here are a little confusing. Be sure to start each paragraph with a general sentence that indicates what you found. Then, discuss each of your results that provides evidence for your statement, and conclude with a sentence that indicates to us the importance of these results. It feels like there are too many concepts included in each paragraph, and the writing is a bit confusing throughout.

**Response**: We appreciate your comment regarding the discussion section. As you suggested, in the revised manuscript, we slightly restructured the paragraphs in the discussion section. We revised each of following paragraphs so that it discusses one specific point each time.

**16. **Last paragraph “However, there are limitationS” (missing s).

 Response: Thank you very much for your comment on the typo. We corrected the point in the revised manuscript.

**17.** These items all feel a bit too similar – Each asks if morality can or cannot be “improved.” Did you consider other items with different word choices? For example, even “You can always become a more moral person with better character.” Or “It is possible to grow in your character and morality.” These items are so similar that it feels hard to argue any differences between them.

 Additionally, if possible it’s always best to include simpler words over more complicated ones. For instance “substantially” could easily be “a lot” and “considerably” could also be “a lot.” More complex words tax the participants a bit more, and may make the sentences more difficult to digest, especially for those with lower readings levels or from less educated backgrounds.

**Response**: We appreciate your comments regarding the items used in our measure. Yes, we agree with you that some words used in the items are somehow complex to be easily understood by younger participants. So, we also think that such items may need to be modified if the measure is to be administrated among younger populations. Also, we also acknowledged that some words (e.g., “improve”) were repeatedly used in multiple items. Since we intended to keep the consistency with the original measures that we referred to (e.g., Dweck’s general growth mindset measure, the Korean version of the MGM measure), we ended up with using such terms in our measure. We explained these points in the limitation section:

*“Fifth, the items used in the MGM measure could be revised particularly when being administered among younger populations. We decided to use the current wordings to maintain consistency with the Korean version of the MGM measure and the Implicit Theory Measure, which constituted the basis of our measure. However, to make the measure more applicable to younger populations, some complex words (e.g., “substantially,” “considerably”) could be replaced with simpler words (e.g., “a lot”). Finally, since several items in the measure might seem to be similar, the words could be revised in future studies, particularly those focusing on children or young adolescents.”*

**18. **There are a few places where the writing could be condensed or the use of alternate terms would improve ease of reading. For instance, an example of condensing would be: in participants, “Participants were recruited from an undergraduate subject pool. The pool consisted of students who were enrolled in educational psychology classes” could be condensed to “Participants were recruited from students enrolled in educational psychology classes.” A for instance of somewhere where alternate terms would be benefitial would be in: Results, “First, all consistency indicators indicated…” Instead of using “indicator/indicated” here I would recommend “First, all consistency indicators revealed…” Or, in the spirit of condensing/being more specific, I might suggest: First, the measure demonstrated at least acceptable reliability according to both cronbach’s alpha values and test-retest reliability.”

 Look for these types of instances throughout the paper with an eye towards condensing repetitive language and becoming more specific.

**Response**: We sincerely appreciate your suggestions regarding the brevity of the manuscript. We agree with you that increasing the brevity is essential to enable potential readers to better understand the overall theme of our manuscript while saving their time. Thus, we edited the whole manuscript during the current revision process. In addition, we revised the manuscript as an effort to minimize repeating to use the same words in the multiple places.

# Peer review: Reviewer response for version 1

**DOI:** 10.5256/f1000research.25564.r62240

**Published:** 2020-04-27

**Authors:** Michael T. Warren

**Affiliations:** 1 Human Early Learning Partnership, University of British Columbia, British Columbia, Canada; 2 Psychology Department, Western Washington University, Bellingham, WA, USA

Han and colleagues examined the psychometric properties of the English version of a growth mindset measure in the moral domain. Moral growth mindset (MGM) may prove to be a useful motivational construct in the scientific study of morality in general, and moral development in particular. I believe the authors present sufficient initial evidence of the validity and reliability of their measure, marking an important step in the scientific study of MGM in English-speaking populations. 

 I see three major issues with the paper in its current form, and I would encourage the authors to revise their manuscript in light of my suggestions.

 First, my biggest concern is the use of phrases such as “improve one’s morals” and “improve your morals.” The former occurs in the paper’s conceptual framing (p. 3) and the latter appears in each of the MGM scale’s four items (p. 4). The issue with these phrases is that improving one’s morals seems to deviate conceptually from improving one’s morality. One might improve their morals by setting new (or higher) moral standards for themselves, yet they may fail miserably in living up to their moral values. By contrast, improving one’s morality involves actually becoming a better person, and this, I believe, is the construct the authors intended to measure. Since in my view the items miss the target to some extent, I have indicated that the work is only “partly” technically sound. Unfortunately, I don’t think much can be done about this issue at this point, but at a minimum I would recommend that the authors either provide an argument for the use of "moral" rather than "morality" in their scale, or identify this as a limitation of their scale. In addition, they might choose to argue that this concern is assuaged by the scale’s strong evidence of convergent validity with other measures of morality.

 Second, I think the CFA results need to be communicated more fully, and that is why I have indicated that the statistical analyses and their interpretations are only “partly” appropriate. On p. 4, I certainly understand consulting previous studies (e.g., Han *et al.*, 2018), but data from the current (i.e., English) study should be given primary importance in refining the English scale. I recommend reporting the factor loadings from the original CFA (i.e., before Items 1 and 2 were removed), so readers can evaluate whether removing these items was justified on empirical grounds. On a related note, I think it would be appropriate to acknowledge the small factor loading (-.39) for the reverse-scored item in Study 2.  

 Third, I think the introduction to Study 2 (p. 5) should be expanded considerably. It would be very helpful to provide a brief rationale for why the selected constructs were chosen for convergent and discriminant validity testing. In addition, it would be helpful to specify hypotheses concerning the strength and direction of the associations between MGM and other constructs (i.e., with which constructs does MGM have strongest and weakest theoretical ties?), and why. The discussion currently states that the observed associations were “as hypothesized,” but no hypotheses were specified in the lead-up to Study 2. I also found myself wondering why Chiu *et al.* ^1^ (1997)’s original 3-item English MGM measure was not included for convergent and incremental validity testing.  

 Minor comments:

- Page 3: My understanding is that growth mindset generally concerns one’s beliefs about the malleability of one’s own (and others’) qualities. Thus, it seems a little bit too generic to define growth mindset as believing “it is possible to improve aspects of one’s life.” There are aspects of a person’s life (e.g., what kind of work they do; where they live, etc.) that are not qualities of their personhood. I suggest the authors consider revising their opening definition of growth mindset. 
- Page 3: It’s not clear to me how allowing participants to define “moral” and “character” necessarily allows them to do so “without bias.” Instead, I think it would be more accurate to say that the approach taken leaves it up to participants to interpret "moral" and "character" according to their own subjective understandings of those terms. (Note that this approach makes no claim that participants’ understandings are “without bias.”)
- Pages 3 and 5: I suggest the authors change the “Participants” heading to “Participants and procedures.”
- Page 4: I suggest confirming that three IRB approvals were needed for just two studies. 
- Pages 4-5: I suggest referring to model fit and reliability indices as either “indices” or “indexes,” rather than “indicators.” Given that CFA was involved, readers may assume “indicators” refers to measured variables loading onto latent factors.
- Page 5 (last paragraph of Study 1): I would like to suggest an alternative explanation as to why Items 1 and 2 (presumably) had lower factor loadings. These two were the only items to convey morality/character as dispositional (e.g., "You have a certain morality and character..."; "Your morality and character are something about you..."). By contrast, all items measured malleability beliefs, including the retained reverse-scored item (“To be honest, you can’t really improve your morals and character.”). My understanding is that a growth mindset is anchored in malleability beliefs, and having a growth mindset does not preclude the belief in moral dispositions (e.g., with effort I can become a more consistently/dispositionally honest person). In other words, perhaps the reason why Items 1 and 2 presumably had lower factor loadings was because they strayed somewhat from the core of the growth mindset construct (i.e., malleability beliefs), rather than because they used the vague qualifier, “much.” Just some food for thought.
- Page 5 (Participants section): Much of the first two paragraphs in this section is redundant with the procedures described in Study 1. The authors may wish to simply state that the same recruitment procedures were used as described in Study 1.
- Page 5: I would strongly urge the authors to omit the term, “marginally correlated” in relation to MGM’s association with the bDIT. Once a threshold for statistical significance has been set (e.g., .05), a finding is either statistically significant or non-significant. Correlations with p-values between .05 and .10 are non-significant. 
- Page 6 (Table 3): I suggest indicating where Cronbach alphas are reported (i.e., on the diagonal).
- Page 6: More information on the potential utility of the MGM measure for understanding moral development would be a nice selling point for the scale. For example, this scale makes it possible to test whether MGM moderates the efficacy of moral education and social emotional learning interventions. The scale would also be an important outcome measure in examining how to nurture MGM (e.g., through process praise, teaching about neuroplasticity, etc.).  
- Page 6: It is not yet clear why the authors would like to have conducted CFAs for the other measures. I would suggest they either drop this piece or further explain why additional CFAs would be desirable if the sample were large enough.
- Page 6: I think more explanation is needed as to why testing measurement invariance would be helpful. For example, the authors might say that examining measurement invariance across diverse groups of people (e.g., political conservatives vs. liberals; young adults vs. older adults) would help evaluate whether the scale—which leaves it up to participants to interpret morality and character in the item stems—in fact measures the same thing for groups who may use different underlying folk conceptions of morality.

Is the work clearly and accurately presented and does it cite the current literature?

Yes

If applicable, is the statistical analysis and its interpretation appropriate?

Partly

Are all the source data underlying the results available to ensure full reproducibility?

Yes

Is the study design appropriate and is the work technically sound?

Partly

Are the conclusions drawn adequately supported by the results?

Yes

Are sufficient details of methods and analysis provided to allow replication by others?

Yes

Reviewer Expertise:

Developmental Psychology, Moral Development

I confirm that I have read this submission and believe that I have an appropriate level of expertise to confirm that it is of an acceptable scientific standard, however I have significant reservations, as outlined above.

## References

[1] : Implicit theories and conceptions of morality. *Journal of Personality and Social Psychology*.1997;73(5) : 10.1037/0022-3514.73.5.923 923-940 10.1037/0022-3514.73.5.923

# Response

**Published:** 2020-05-06

**Authors:** Hyemin Han

**Affiliations:** University of Alabama, USA

Dear Dr. Warren,

 We sincerely appreciate your comments and suggestions on our manuscript. We found that they are very constructive and information. While revising our manuscript, we have done our best to address the concerns that you mentioned in your review report. Please find our responses to your comments below. Thank you very much for your time and consideration.

 Best,

 Hyemin Han (Corresponding author)

**Responses**

**1. **First, my biggest concern is the use of phrases such as “improve one’s morals” and “improve your morals.” The former occurs in the paper’s conceptual framing (p. 3) and the latter appears in each of the MGM scale’s four items (p. 4). The issue with these phrases is that improving one’s morals seems to deviate conceptually from improving one’s morality. One might improve their morals by setting new (or higher) moral standards for themselves, yet they may fail miserably in living up to their moral values. By contrast, improving one’s morality involves actually becoming a better person, and this, I believe, is the construct the authors intended to measure. Since in my view the items miss the target to some extent, I have indicated that the work is only “partly” technically sound. Unfortunately, I don’t think much can be done about this issue at this point, but at a minimum I would recommend that the authors either provide an argument for the use of "moral" rather than "morality" in their scale, or identify this as a limitation of their scale. In addition, they might choose to argue that this concern is assuaged by the scale’s strong evidence of convergent validity with other measures of morality. 

**Response**: Thank you very much for your comment regarding the use of the terms in our study. In the revised manuscript, we added a paragraph describing why we decided to use “morals” instead of “morality” in our measure. The point is that we intended use the term to maintain the consistency with the prior study.

*“Finally, since Chiu et al. used terms related to specific morals and characteristics in their original three-item subscale (e.g., “A person’s moral character,” “whether a person is responsible and sincere,” “a person’s moral traits”), we decided to use “morals and character” in order to stay consistent with the construct they were measuring. That is, rather than measuring participants’ malleability beliefs about the overarching system of values they have, we wanted to measure malleability beliefs regarding individual morals, as did the original measure. Doing so may increase the chance for interventions since if people want to become a better person (improve their morality) they may need to believe that their values (morals) can be improved.”*

**2. **Second, I think the CFA results need to be communicated more fully, and that is why I have indicated that the statistical analyses and their interpretations are only “partly” appropriate. On p. 4, I certainly understand consulting previous studies (e.g., Han *et al.*, 2018), but data from the current (i.e., English) study should be given primary importance in refining the English scale. I recommend reporting the factor loadings from the original CFA (i.e., before Items 1 and 2 were removed), so readers can evaluate whether removing these items was justified on empirical grounds. On a related note, I think it would be appropriate to acknowledge the small factor loading (-.39) for the reverse-scored item in Study 2.   

**Response**: We appreciate your comment regarding how to report results from CFA. Following your suggestion, we added a supplementary table that demonstrates the factor loadings in 6-item and 5-item models. As you can see, Item 1 and Item 2 showed the lowest standardized factor loadings in the 6-item and 5-item models, respectively. In the revised manuscript, we mentioned the point that they were excluded from the measure due to their lowest standardized factor loadings.

 Table S1

 Factor loadings from the CFA of the six- and five-item models in Study 1

 Six-item model

 Five-item model

 Item

 Unstandardized

 Standardized

 Unstandardized

 Standardized

**You have certain morals and character, and you can’t really do much to improve it.**

**-.71**

**-.58**

**-**

**-**

**Your morals and character are something about you that you can’t improve very much.**

 .70

 -.67

**-.61**

**-.58**

 No matter who you are, you can significantly improve your morals and character.

 .64

 .62

 .68

 .65

 To be honest, you can’t really improve your morals and character.

 -.86

 -.86

 -.81

 -.81

 You can always substantially improve your morals and character.

 .66

 .69

 .72

 .75

 You can improve your basic morals and character considerably.

 .80

 .'.83

 .85

 .88

 Note: Bolded items (Items 1 and 2) were excluded from the finalized MGM measure.

*“In the supplementary table in Underlying data, we presented factor loadings for the six-item and five-item models. In the six-item model, Item 1 showed the lowest standardized factor loading, identical to what was reported in Han et al. (2018) 4. After excluding Item 1, Item 2 showed the lowest standardized loading in the five-item model, so we removed this item accordingly.”*

 Moreover, we acknowledge the slightly low factor loading in Study 2:

*“However, it should be acknowledged that Item 4 showed a slightly lower factor loading in Study 2 compared with Study 1, although the overall model fit indices were excellent. This point might need to be tested in future studies with more samples.”*

**3. **Third, I think the introduction to Study 2 (p. 5) should be expanded considerably. It would be very helpful to provide a brief rationale for why the selected constructs were chosen for convergent and discriminant validity testing. In addition, it would be helpful to specify hypotheses concerning the strength and direction of the associations between MGM and other constructs (i.e., with which constructs does MGM have strongest and weakest theoretical ties?), and why. The discussion currently states that the observed associations were “as hypothesized,” but no hypotheses were specified in the lead-up to Study 2. I also found myself wondering why Chiu *et al.* (1997)’s original 3-item English MGM measure was not included for convergent and incremental validity testing.  

**Response**: Thanks a lot for your suggestion regarding the expansion of the Study 2 introduction. Following your suggestion, in the revised manuscript, we elaborated the rationale regarding how the additional construct used in our study were selected with citations. Furthermore, in the methods section, per additional measurement, we explained the direction and effect size of the hypothesized correlation.

*“We selected several moral and positive psychological measures to test the convergent and divergent validity of the MGM measure. We employed the Implicit Theory Measure 1, which measures domain-general growth mindset and constitutes the basis of the MGM measure, to test convergent and discriminant validity. For the selection of moral psychological measures, we referred to recent articles about psychological constructs that significantly predict prosocial and civic behavior 31. They proposed moral judgment 18, 19, moral emotion (empathy) 20, and moral identity 21 as fundamental constructs in moral functioning. We also employed the Propensity to Morally Disengage Scale to examine whether the MGM showed negative correlation with moral disengagement 22 since Han et al. (2018) 4 reported that MGM promotes moral engagement. In addition to the aforementioned moral psychological measures, we used the Claremont Purpose Scale as a way to examine one’s positive development in terms of flourishing 23, given that purpose has been regarded as a possible moral virtue for eudemonic wellbeing 32.*

*In general, according to the previous studies that examined the relationship between growth mindset, positive psychological indicators, and antisocial tendency (e.g., 24– 26), we hypothesized that the sizes of correlation coefficients between MGM and other indicators, except the general growth mindset, would be between .10 (small) and .30 (medium). We discussed further details regarding the hypothesized effect size of each measure in the following sections.”*

 We agree with you that employing Chiu et al.’s original measure in the present study would be beneficial. However, we did not consider doing so because our measure was originally Based on Dweck’s updated six-item measure for general growth mindset. In the limitation section, we acknowledged your point for reader’s information.

*“Fourth, we did not employ Chiu et al.’s (1997) 5 original measure, which could be informative while conducting the convergent validity check, although our measure was based on Dweck’s (2000) 1 updated six-item general growth mindset measure.”*

**4.** Page 3: My understanding is that growth mindset generally concerns one’s beliefs about the malleability of one’s own (and others’) qualities. Thus, it seems a little bit too generic to define growth mindset as believing “it is possible to improve aspects of one’s life.” There are aspects of a person’s life (e.g., what kind of work they do; where they live, etc.) that are not qualities of their personhood. I suggest the authors consider revising their opening definition of growth mindset.  

**Response**: We appreciate your suggestion regarding the introduction. We revised the introduction for a better definition of the growth mindset:

*“Growth mindset refers to the belief that it is possible to improve one’s abilities and qualities, such as intelligence or personality 1 . These individuals believe that this can be done through effort and learning, which helps fosters motivation. Higher motivation for those with a growth mindset is encouraged through having attitudes such as viewing hardships as a chance to work harder  rather than an indication of  failure, and striving for success due to genuinely wanting to learn instead of being concerned with how others view them 2”*

**5. **Page 3: It’s not clear to me how allowing participants to define “moral” and “character” necessarily allows them to do so “without bias.” Instead, I think it would be more accurate to say that the approach taken leaves it up to participants to interpret "moral" and "character" according to their own subjective understandings of those terms. (Note that this approach makes no claim that participants’ understandings are “without bias.”) 

**Response**: We appreciate your comment regarding the use of the terms in our study. In the revised manuscript, we updated our explanation regarding the terms as per your comment:

*“Thus, we used “morals and characters” in order for participants to be able to define the terms based on their own experiences and understanding.”*

**6.** Pages 3 and 5: I suggest the authors change the “Participants” heading to “Participants and procedures.” 

**Response**: Thanks a lot for your suggestion regarding the subsection. In the revised manuscript, following your and Dr. Mangan’s suggestions, we moved contents regarding the study procedures to a new subsection, “procedures.”

**7.** Page 4: I suggest confirming that three IRB approvals were needed for just two studies. 

**Response**: Thank you for your comment regarding the IRB numbers. In the revised manuscript, we clearly stated which IRB protocols are relevant to which specific study.

**8.** Pages 4-5: I suggest referring to model fit and reliability indices as either “indices” or “indexes,” rather than “indicators.” Given that CFA was involved, readers may assume “indicators” refers to measured variables loading onto latent factors. 

**Response**: We appreciate your comment regarding the use of the term. In the revised manuscript, as per your comment, we used “indices” in lieu of “indicators” while addressing CFA.

**9.** Page 5 (last paragraph of Study 1): I would like to suggest an alternative explanation as to why Items 1 and 2 (presumably) had lower factor loadings. These two were the only items to convey morality/character as dispositional (e.g., "You have a certain morality and character..."; "Your morality and character are something about you..."). By contrast, all items measured malleability beliefs, including the retained reverse-scored item (“To be honest, you can’t really improve your morals and character.”). My understanding is that a growth mindset is anchored in malleability beliefs, and having a growth mindset does not preclude the belief in moral dispositions (e.g., with effort I can become a more consistently/dispositionally honest person). In other words, perhaps the reason why Items 1 and 2 presumably had lower factor loadings was because they strayed somewhat from the core of the growth mindset construct (i.e., malleability beliefs), rather than because they used the vague qualifier, “much.” Just some food for thought. 

**Response**: Thanks a lot for the alternative explanation of the lower factor loadings of items 1 and 2. We added such an alternative explanation in the revised manuscript for readers’ information:

*“In addition, as another possibility, items 1 and 2 are more likely about entity beliefs, not malleability beliefs that constitute the basis of growth mindset. These items contain some words perhaps related to entity beliefs (e.g., “certain morals and characters...,” “something about you…”), so they might not directly measure the core of the growth mindset construct and showed lower factor loadings compared to the other items.”*

**10.** Page 5 (Participants section): Much of the first two paragraphs in this section is redundant with the procedures described in Study 1. The authors may wish to simply state that the same recruitment procedures were used as described in Study 1. 

**Response**: Thank you very much for your suggestion for the brevity of our manuscript. We shortened the redundant part in Study 2 as per your suggestion.

**11. **Page 5: I would strongly urge the authors to omit the term, “marginally correlated” in relation to MGM’s association with the bDIT. Once a threshold for statistical significance has been set (e.g., .05), a finding is either statistically significant or non-significant. Correlations with p-values between .05 and .10 are non-significant. 

**Response**: Thanks a lot for your comment about the use of the term, “marginally correlated.” We agree with you that the use of the term is somehow inappropriate, so in the revised manuscript, we changed the part about interpreting the finding from correlation analysis:

*“The effect size of the correlation coefficient between MGM and bDIT was small as predicted, but the correlation was non-significant (p = .08).”*

**12.** Page 6 (Table 3): I suggest indicating where Cronbach alphas are reported (i.e., on the diagonal).

**Response**: We appreciate your suggestion. We added a brief description about where alphas were reported:

*“Cronbach αs are also reported (on the diagonal).”*

**13.** Page 6: More information on the potential utility of the MGM measure for understanding moral development would be a nice selling point for the scale. For example, this scale makes it possible to test whether MGM moderates the efficacy of moral education and social emotional learning interventions. The scale would also be an important outcome measure in examining how to nurture MGM (e.g., through process praise, teaching about neuroplasticity, etc.).   

**Response**: Thanks a lot for your suggestion about the elaboration of the potential utility of the measure. In the introduction, we briefly mentioned how the measure could be used in moral education:

*“Additionally, if moral growth mindset motivates people to learn how to become more moral, as previous research suggests, then it is important for moral educators to have a tool to assess the malleability beliefs students have related to their morals. For example, if moral educators are able to identify that some students have a fixed mindset related to their morals, then an appropriate starting point may be to provide them with evidence that it is possible to improve moral character throughout one’s life.”*

**14. **Page 6: It is not yet clear why the authors would like to have conducted CFAs for the other measures. I would suggest they either drop this piece or further explain why additional CFAs would be desirable if the sample were large enough. 

**Response**: We appreciate your comment regarding the CFA of additional measures. We dropped the part as per your comment since it was not essential in our study.

**15. **Page 6: I think more explanation is needed as to why testing measurement invariance would be helpful. For example, the authors might say that examining measurement invariance across diverse groups of people (e.g., political conservatives vs. liberals; young adults vs. older adults) would help evaluate whether the scale—which leaves it up to participants to interpret morality and character in the item stems—in fact measures the same thing for groups who may use different underlying folk conceptions of morality. 

**Response**: Thank you very much for your suggestion. We clarified the point in the revised manuscript:

*“To address this issue, measurement invariance test would be a way to examine whether the MGM measure, which allows participants to interpret “morals” and “character” by themselves, measures the same construct across different groups who may use different underlying folk conceptions of morals and character.”*
